# Structural characterization of genomes by large scale sequence-structure threading: application of reliability analysis in structural genomics

**DOI:** 10.1186/1471-2105-5-101

**Published:** 2004-07-26

**Authors:** Artem Cherkasov, Shannan J Ho Sui, Robert C Brunham, Steven JM Jones

**Affiliations:** 1 Division of Infectious Diseases, Faculty of Medicine, University of British Columbia, Vancouver, British Columbia, Canada; 2 Department of Molecular Biology and Biochemistry, Simon Fraser University, Burnaby, British Columbia, Canada; 3 University of British Columbia Center for Disease Control, Vancouver, British Columbia, Canada; 4 Genome Sciences Centre, British Columbia Cancer Agency, Vancouver, British Columbia, Canada

## Abstract

**Background:**

We establish that the occurrence of protein folds among genomes can be accurately described with a Weibull function. Systems which exhibit Weibull character can be interpreted with reliability theory commonly used in engineering analysis. For instance, Weibull distributions are widely used in reliability, maintainability and safety work to model time-to-failure of mechanical devices, mechanisms, building constructions and equipment.

**Results:**

We have found that the Weibull function describes protein fold distribution within and among genomes more accurately than conventional power functions which have been used in a number of structural genomic studies reported to date.

It has also been found that the Weibull reliability parameter *β *for protein fold distributions varies between genomes and may reflect differences in rates of gene duplication in evolutionary history of organisms.

**Conclusions:**

The results of this work demonstrate that reliability analysis can provide useful insights and testable predictions in the fields of comparative and structural genomics.

## Background

Recent advances in networks theory have demonstrated a key role of uneven distributions occurring in many natural processes. It has been found that seemingly unrelated systems such as economic, professional, sexual and social networks, airline routing, power lines connections, language networks and internet hyperlinks all exhibit a power law decay of the cumulative distribution *P*_*x *_≈ *x*^-*γ*^, where *x *is the number of links connected to each network node and *γ *is the value of the exponent typically varying in the range of 2–3 [1]. The heterogeneous architecture of scale-free networks imparts a robustness and error-tolerance from random perturbation and is often viewed as a possible common blueprint for naturally occurring large-scale networks. The critical role of the power law distribution has also been acknowledged in many areas of life sciences: metabolic and other cellular networks, proteins interaction maps, brain cellular organization, food and ecological webs all have been described as scale-free systems. It would be fair to say that the advances in the scale free network studies have revitalized the original Pareto's inequality law introduced more then a century ago [2].

The applicability of the scale free networks has been examined in numerous structural genomics studies. It has been proposed that the genomic occurrence of protein families, superfamilies and folds can follows an asymptotic power law:

*SDF(GO) *= *aGO*^-*b *^    (1)

, where *SDF(GO) *is survival distribution function of genomic occurrence *GO *of a certain protein family, superfamily and fold. These findings have laid the foundation for characterizing the evolution of the protein universe in terms of a growing scale-free system in which individual genes are represented as nodes of a propagating network [3-7].

In our previous work [9], we have used the large-scale sequence-structure threading to assign protein folds to 33 genomes from all three superkingdoms of life. It has been found that more then 60% of the studied eukaryotic, 68% of archaeal and 70% of bacterial proteomes could be assigned to defined protein folds by threading. The estimated results have been used to analyze the distribution of protein architectures, topologies and domains (or homologous superfamilies according to the CATH classification [8]). Thus, we have found that the frequencies of genomic occurrence of assigned protein domains (homologous superfamilies) and topologies can be described by a power function (1) with moderate accuracy. According to the formalism of network theory, such a power law representation of the cumulative distribution of node connections governs a scale-free character of the system [10]. At the same time we have noted that the values of the power exponent *b *estimated in the study generally fall below the 2–3 range typical for scale-free systems (analogous observations could also be noted in a number of similar investigations [3-5]). Table 1 (see Additional file 1) features the estimated parameters *a *and *b *along with the corresponding correlation coefficients *r*^2 ^reflecting the goodness of fit of experimental data with the logarithmic linear plots (1) (Table 1 also reflects the total number of the analyzed ORF-s in each genome and the corresponding number of proteins for which the THREADER has confidently assigned certain fold).

The established lowered values of the power exponent and modest accuracy of the power law dependences (1) encouraged us to seek alternative approaches to more accurately describe protein folds distributions.

## Results

### Weibull (reliability) analysis

The Weibull distribution is a general-purpose statistical function defined within Extreme Value Theory [11] and widely used in reliability engineering to model material strength, durability of electronic and mechanical components or equipments. In the most common case the probability density distribution is described by a two-parameter Weibull distribution


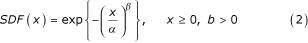


, where *α *is a scaling factor and *β *is a shape parameter also known as the slope [12].

The Weibull analysis operates on life data, *i.e *it utilizes time-to-failure (or time under the testing stress) to assess the reliability of a system and to forecast its stability through parameters of the characteristic life span *α *and shape *β*. A typical Weibull experiment is based on application of disruptive stress to multiple samples representative of the population until the tested objects achieve a state of failure and produce time-to-failure numbers. The corresponding time-to-failure values form heterogeneous Weibull distributions described by (2).

### Application of Weibull function to genomic analysis

The distribution of protein folds in a genome can be viewed much like the behavior of a mechanical system under disruptive testing. It is feasible to stipulate that the increase of genomic abundance of any protein fold occurs under evolutionary pressure. Some folds are able to expand their genomic occurrence over a course of evolution others have higher probability to be lost through genetic drift and other random events, *i.e. *to fail. Considering these analogies, we anticipated that the Weibull logistic can provide some natural explanations for highly heterogeneous abundance of protein folds in genomes. To test this hypothesis we used two independent approaches to examine whether the genomic occurrence of protein topologies and domains can indeed be adequately described by the Weibull function.

First of all, we employed the maximum likelihood (ML) method [13] to fit the survival distribution function *SDF(x) *of the genomic occurrences of protein topologies and homologous superfamilies into the Weibull dependence (2). The corresponding Weibull shape parameters have been established by solving the equation 
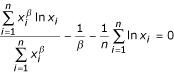
 while the scaling factors have been calculated as 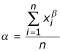.

**Figure 1:**
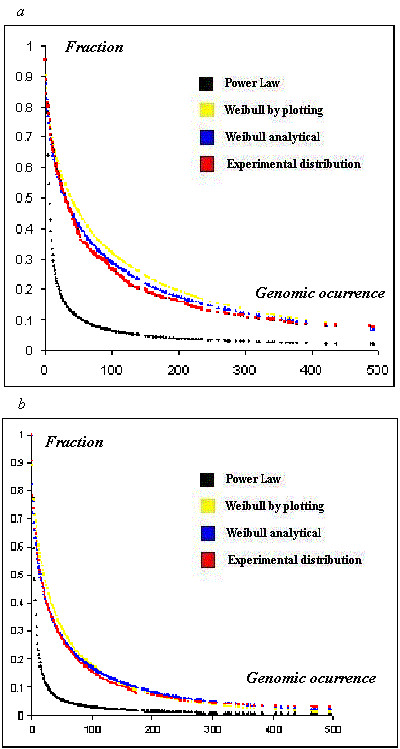
Observed and recalculated survival distribution functions of genomic occurrences of protein topologies (a) and domains (b) among all the studied genomes combined.

The ML method allowed very accurate description of the distribution of protein folds among the genomes. Figures 1a and 1b feature the survival distributions of CATH topologies and homologous superfamilies among all the studied genomes in combined (these experimental (observed) data curves are marked in red). On the same graphs we have plotted the *SDF(GO) *parameters reproduced within equation (2) through *α *and *β *values estimated by the ML approach. It is obvious that these computed blue curves labeled as 'Weibull analytical' resemble the experimental distributions (marked in red) very precisely.

The corresponding *α *and *β *values estimated by the ML approach have been collected into Table 2 (see Additional file 2).

The second way of examining applicability of the Weibull function (2) was based on notion that the double logarithmic transformation of the *SDF(x) *leads to the equation of a straight line:

log(- log(*SDF*(*x*)) = *β *log(*x*) - *β *log(*α*)     (3)

We performed the transformation (3) on the experimental *SDF(GO) *data to estimate the Weibull coefficients *α *and *β *and squared correlation coefficients *r*^2 ^which all have also been collected into Table 2 (marked as 'Weibull by plotting').

The values of the estimated squared correlation coefficients *r*^2 ^from Table 2 demonstrate very high accuracy of the linear dependences (3) established from the survival distributions of CATH folds in the studied genomes. These parameters also allow comparing the accuracy of double logarithmic dependences (3) with accuracy of simple logarithmic dependences derived from the power law model (1):

*log*(*SDF*) = *a *- b*log(*GO*)     (4)

As it has been mentioned earlier, the dependences (4) have been estimated for the *SDF(GO) *functions for individual genomes, superkingdoms and for the combined set of proteins. The comparison of *r*^2 ^values from Table 2 and Table 1 established from the linear functions (3) and (4) respectively, reveals that for all studies cases (individual genomes, superkingdoms, total dependences) the statistical quality of Weibull dependences (3) is much better than of power law function (4). Figures 1a and 1b feature the Weibull distributions estimated by plotting (double logarithmic transformation) which reproduce the experimental *SDF(x) *curves with remarkable accuracy. Apparently, the distributions calculated from (3) (labeled as 'Weibull by plotting') are much closer to the experimental distributions than the power law curves (labeled as 'power law') computed within the conventional power function (1). Apparently, that the Weibull distributions established by the double logarithmic representations (4) (marked on Figure 1 'Weibull by plotting') are very close to those calculated by the ML method ('Weibull analytical'). It should be mentioned, however, that despite close resemblance between the Weibull distributions established by the analytical ML method and the 'double logarithmic' approach, the corresponding values of *α *and *β *parameters from Table 2 differ (due to the different data fitting algorithms employed by two methods) and the preference should, perhaps, be given to more stringent ML-derived data.

### Characteristic conditions for the Weibull distribution

Although the estimated statistical criteria clearly demonstrate the suitability and superiority of a Weibull function over a power function in describing protein fold distributions, we decided to examine several additional criteria characteristic of the Weibull distribution. As it has been suggested by Romeu [14] there are four such characteristic properties immanent for the Weibull function.

#### The double logarithmic plot of life data (also called 'a Weibull paper') should be linear

**Figure 2:**
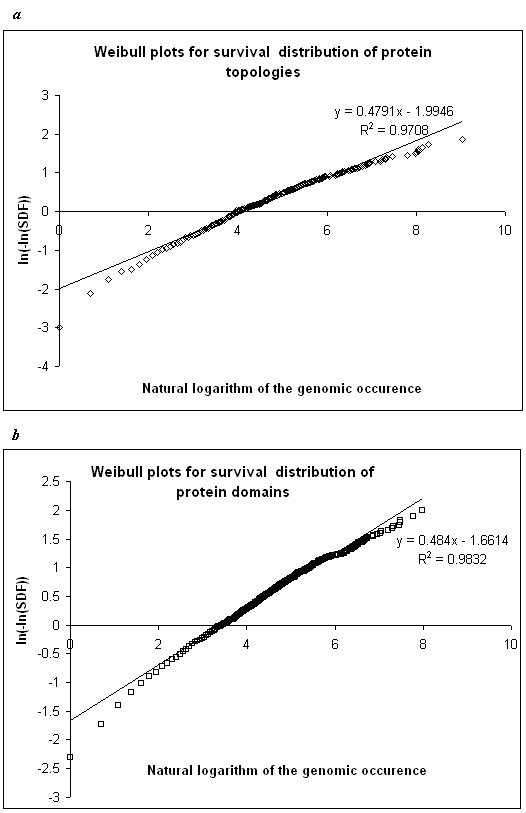
Weibull plots for survival distributions of genomic occurrences of protein topologies (2) and domains (2) among the studied genomes.

As it can be seen from Table 1 the estimated *r*^2 ^values from the columns marked as 'Weibull by plotting' are all contained within the range ~*0.95*–*0.98 *what demonstrates that the 'Weibull papers' do indeed describe protein folds distribution in the studied genomes with high accuracy. Figures 2a,2b feature the 'Weibull papers' for the distribution of protein topologies and domains among all the studied species and illustrate that deviations from linearity are very insignificant.

#### The slope of the 'Weibull paper' is an alternative estimator of *β*

The data from Table 2 demonstrate that the estimated slopes of the 'Weibull papers' are very close to the values of *β *derived by analytical maximum likelihood approach.

#### The *x*^*β *^transformation should yield an exponential distribution with mean *α*^*β*^

The genomic occurrences of protein topologies and domains in the genomes and superkingdoms have been transformed into *GO*^*β *^distributions through the power factors *β*. The exponential character of the resulting distribution has been examined by several statistical tests and in all cases has been confirmed. The observed medians of the exponential distributions *GO*^*β *^accumulated in Table 3 (see Additional file 3) demonstrate strong correlations with the calculated *α*^*β *^values.

#### Characteristic life *α *of the Weibull distribution lies approximately at the 63% of the population

The values of the Weibull characteristic life at 63% of distributions have been calculated and collected in Table 3. It is obvious that these parameters closely match values *α *defined by plotting.

Thus, all four specific criteria studied indicate that the genomic occurrence of protein topologies and domains can be characterized as true Weibull distributions. To support this notion further we have also considered another important property of the he Weibull distribution – the dependence of its median (*MDN*) from shape and scale parameters [13]:


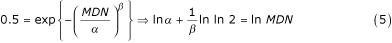


**Figure 3:**
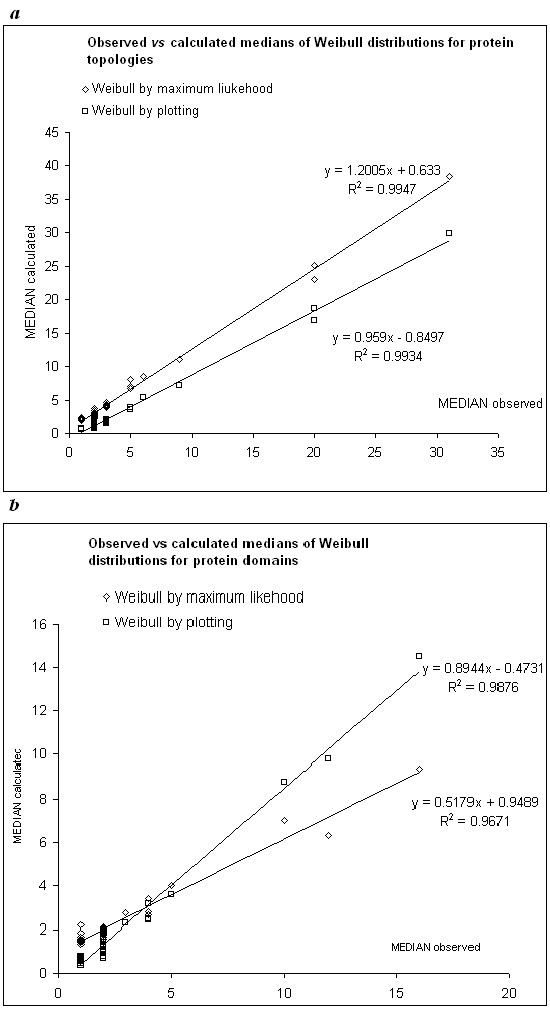
Observed vs. calculated medians of the Weibull distributions established by the maximum likelihood and plotting methods for protein topologies (a) and domains (b).

To assess the applicability of this condition, we calculated Weibull medians using sets of *α *and *β *parameters – estimated by graphical (double logarithmic transformation) and analytical (ML) approaches. The corresponding '*MDN Calctd*' values have been collected into Table 3 along with the observed medians of the Weibull distributions (marked '*MDN Obsrvd*'). The estimated high quality linear dependences between the theoretical and observed medians are present on figures 3a and 3b for topologies and domains distributions respectively. The graphics demonstrate that calculated and observed median values are virtually the same what unanimously confirms validity of the condition (5).

Thus, multiple independent tests have demonstrated that occurrence of protein folds in genomes obey the Weibull distribution and therefore can be interpreted in terms of the reliability theory what can provide additional insight into folds evolution.

## Discussion

### Interpretation of the Weibull parameters

The very fact that we were able to assign the Weibull character to the distributions of the CATH protein topologies and homologous superfamilies within genomes ultimately implies that parameters of genomic occurrence can be classified as extreme values. According to the Extreme Values Theory the Weibull distribution will successfully model life systems for which many competing similar failure processes are "racing" to failure and the first to reach it produces the observed failure time [15]. In regard to genomic occurrence this may suggest that protein folds increase their genomic occurrence in a competitive manner and that those folds having a greater potential to duplicate, will continue to duplicate at the cost of less abundant folds which may ultimately disappear from genome.

On another hand, according to reliability theory a Weibull distribution with *β *> 1 characterizes a life system that increasingly deteriorates. If the shape parameter is smaller then unity (*β *<1), there is a reliability growth as the failure rate of the system decreases with time [14]. It is not clear at the moment, whether a reliability criterion is directly applicable to protein folds distributions. However, *β *does indeed describe the "skewdness" of the fold distribution, for example *Caenorhabditis elegans *has the lowest calculated value *β *among the studied organisms, whilst this genome has also been characterized for its recent expansion and duplication of several gene families [16]. Presumably, many of these folds are present at lower abundances in other genomes. It could be proposed that such a low *β *(according to the reliability theory characterizing the genome of *C. elegans *as the most stable amongst the studied) may reflect the fact that chances of loosing some lower abundant fold families are lower for *C. elegans *(considering that >70% of the translated ORFs *C. elegans *genome have been covered by the sequence-structure threading we have assumed that the recently duplicate genes are accordingly represented in the results). In this context, the reliability of a proteome can be viewed as its ability to maintain and expand its composition without loss of protein folds.

We can speculate that life systems that enjoy evolutionary success will tend to minimize *β *<1 *i.e. *to have more balanced (less heterogeneous) folds representation in their genomes. The fact that most *β *values presented in Table 2 fall below the unity threshold demonstrates that, in general, the reliability of genome fold composition increases with time, *i.e. *less protein folds reach the failure state (termination of multiplication and, likely, following evolutionary extinction) as an organism evolves.

**Figure 4:**
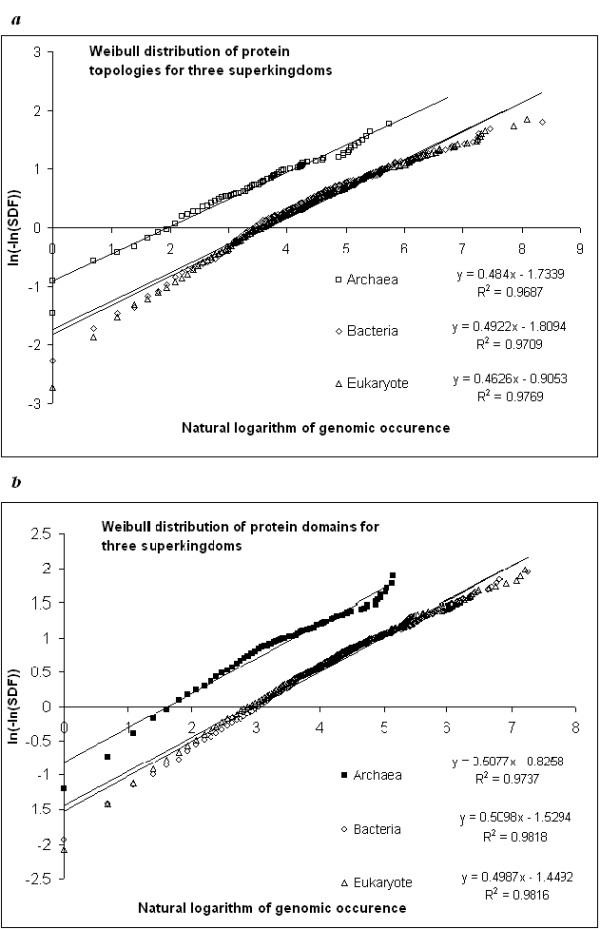
Weibull distributions of protein topologies (a) and domains (b) among three superkingdoms (Archaea, Bacteria and Eukaryote).

Interestingly, little difference is observed has been found between the *β *shape parameters for topologies distributions across the three superkingdoms. All three linear dependences ln(- ln(*SDF*(*GO*))) ~ ln(*GO*) for Bacteria, Eukaryote and Archaea presented on Figures 4a,4b appear very similar.

As it has been already mentioned above, it is difficult to decide at this point whether the observed Weibull character of protein folds distribution can be placed in a larger context. We can only speculate that protein folding preferences may lead to a greater abundance of favorable protein configurations and to extinction of those folds which are less favorable. Such selection may represent a mechanism of evolutionary quest for searching for better protein folds. In any case, the observed phenomenon illustrates the act of natural selection in determination of the protein fold repertoires and that the propagation of protein folds in a genome occurs in a competitive manner, *i.e. *more abundant folds tend to expand their genomic presence even further causing lesser abundant folds to extinct.

It also remains to be seen whether some other properties of genomes and proteomes can also be described by the Weibull statistics. In our studies we plan to use the Weibull approach to examine other distributions such as genomic occurrence of transcriptional promoters and regulatory elements, levels of gene expression and occurrence of protein domains per gene, among others.

Another possible development for the reliability analysis in structural genomics might be to investigate whether the standard libraries of proteins folds themselves can be adequately described by the Weibull function. As it has been stipulated, in the study we have used the CATH standard library of protein folds, which is one of the most accepted and used protein folds classifications. Ii is not unfeasible, that the representation of protein architectures, topologies, homologous superfamilies, *etc *in the CATH can be adequately described by the Weibull law. Thus, it has been previously demonstrated that another widely used folds library – SCOP does indeed obey the power low [4]. Such observations would not necessarily contradict the uneven character of the fold distributions in individual proteomes or superkingdoms as a given protein fold library should reflect the proportion of protein folds occurrence in nature. At the same time, we anticipate that the analysis of the standard fold libraries in terms of the Weibull distributions may bring an additional insight into the field and will be carried out in the near future.

To summarize the current work, it is possible to conclude that the use of the Weibull distribution allows more accurate description of protein topologies and domains distributions within and among genomes than power function used in conventional structural genomic studies. In addition, we were able to establish the Extreme Values relationships for protein folds distributions to demonstrate that the protein fold repertoire of an organism most likely occurs as a result of the competition amongst folds. This may reflect a mechanism of natural selection searching for an optimal protein structures when more evolutionary favorable folds tend to populate the entire genomic space and cause the extinction of lesser favorable protein configurations.

## Conclusions

Use of a Weibull function allows describing cumulative distribution of protein topologies and domains within individual genomes and superkingdoms with higher accuracy compared to the conventional power function used in the related studies. The developed approach may be applied to quantification of the distribution of different properties of genomes and can be particularly useful for assessing and comparing fold distributions between different organisms and possible impact of the "reliability" of organisms due to a higher redundancy in their fold composition.

In general, the results of investigation demonstrate the feasibility and importance of using the reliability analysis to improve the bioinformatics analysis of proteomes.

## Methods

### Assignment of protein folds

The prediction of the protein folds has been conducted using the THREADER2 program [17]. The CATH homologues superfamily representative has been assigned to a given protein sequence if the THREADER2 produced an output above 2.9 for the Z score for the threading energy. After a certain CATH entry has been assigned to a protein sequence, it has also been associated with the corresponding higher level CATH representations: class, architecture and topology.

The translated protein sequences for 33 complete genomes downloaded from the NCBI and ENSEMBL databases have been processed in this manner. The threading computations have been paralleled for processing on a Beowulf cluster consisting of 52 dual processor blades (2 × 1 GHz, 1 G RAM). The automated control was implemented by our own PVM-supporting Perl scripts enabling to distribute and query the individual threading processes over multiple computer servers.

### Survival distribution calculation

After the occurrences of distinct classes, architectures, topologies and homologue family representatives have been established within the individual genomes, superkingdoms and in total, the corresponding survival distributions have been computed. First of all, we have established the counts of protein architectures, topologies and domains (homologues families) with a given genomic occurrence *GO*. At the next step these numbers have been converted into the fractional values. After that the survival distribution functions *SDF(x) *have been computed for genomes, superfamilies and for the combined set of proteins. The *SDF(GO) *numbers have been calculated for each integer *GO *value in the range from 0 to the maximal *GO *estimated within the set (genome/superfamily/total).

### Statistical analysis

The fitting of the *SDF*(*GO*)~*GO *functions has been conducted by the SAS 9.0 statistical package (SAS Inc.). The power law dependences *SDF(GO) *= *aGO*^-*b *^have been analyzed as a logarithmic transforms

Log(*SDF(GO) *= *a *- b*GO

where the fitting has been conducted for the linear function.

The Weibull – like dependences *SDF(GO) *= *exp(-aGO*^*b*^) have been fitted using both non-linear approximation (by maximum likelihood method) and by the linear fitting of the double logarithmic transform:

log(- log(*SDF*(*x*)) = *β *log(*x*) - *β *log(*α*)

The calculation of median valued for the survival distribution has been done by the 'R-project' open source statistical package.

## Supplementary Material

Additional File 1 Parameters of power – law dependences for the survival distribution of genomic occurrences SDF(GO) = a GO^b^. Click here for file

Additional File 2Parameters *α*, *β *and medians (calculated and observed) of Weibull distribution of survival functions of genome occurrences 
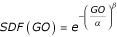
 established by maximum likelihood and plotting methods.Click here for file

Additional File 3 Statistical parameters for 'Weibull papers' for genomic occurrences of protein topologies and domains. Click here for file
