# Supplementary material for: Structural characterization of genomes by large scale sequence-structure threading: application of reliability analysis in structural genomics
Source: BMC Bioinformatics. 2004 Jul 26;5:101. doi: 10.1186/1471-2105-5-101 (PMC499543; doi:10.1186/1471-2105-5-101)
Supplement: Additional File 1 — Parameters of power – law dependences for the survival distribution of genomic occurrences SDF(GO) = a GOb. [file 1471-2105-5-101-S1.doc]

**Table 1. Parameters of power – law dependences for the survival distribution of genomic occurrences SDF(GO) = a GO^b^**.

| ***Organism*** | SK | ORFs | Z>2.9 | **TOPOLOGIES** | | | **DOMAINS** | | |
| --- | --- | --- | --- | --- | --- | --- | --- | --- | --- |
|  |  |  |  | *log(SDF) vs. log(GO)* | | | *log(SDF) vs. log(GO)* | | |
|  |  |  |  | ***a*** | ***b*** | ***r^2^*** | ***a*** | ***b*** | ***r^2^*** |
| *Homo sapiens* | **E** | 30584 | 18413 | 1.35 | -0.90 | 0.925 | -1.17 | 0.921 | 5.37 |
| *Mus musculus* | **E** | 23426 | 11898 | 1.20 | -0.94 | 0.909 | 1.45 | -1.27 | 0.903 |
| *Caenorhabditis elegance* | **E** | 17096 | 11986 | 0.83 | -0.82 | 0.946 | 1.05 | -1.07 | 0.943 |
| *Drosophila melanogaster* | **E** | 14334 | 9407 | 0.90 | -0.89 | 0.941 | 1.09 | -1.17 | 0.927 |
| *Saccharomyces cerevisiae* | **E** | 6298 | 4028 | 0.48 | -0.91 | 0.938 | 0.67 | -1.23 | 0.925 |
| *Plasmodium falciparum* | **E** | 2072 | 991 | 0.33 | -1.12 | 0.877 | -0.08 | -1.27 | 0.945 |
|  |  |  |  |  |  |  |  |  |  |
| *Pseudomonas*  *aeruginosa*  *strain PAO1* | **B** | 5564 | 4104 | 0.55 | -0.94 | 0.953 | 0.79 | -1.29 | 0.919 |
| *Escherichia coli*  *strain CFT073* | **B** | 5378 | 3621 | 0.54 | -0.96 | 0.941 | 0.67 | -1.31 | 0.928 |
| *Escherichia coli*  *strain O157 EDL933* | **B** | 5349 | 3663 | 0.53 | -0.97 | 0.949 | 0.61 | -1.29 | 0.937 |
| *Escherichia coli*  *strain O157* | **B** | 5281 | 3629 | 0.53 | -0.97 | 0.948 | 0.61 | -1.29 | 0.936 |
| *Salmonella typhimurium*  *strain_LT2* | **B** | 4450 | 3185 | 0.32 | -0.93 | 0.952 | 0.51 | -1.27 | 0.933 |
| *Salmonella typhi* | **B** | 4394 | 3097 | 0.34 | -0.94 | 0.956 | 0.49 | -1.28 | 0.934 |
| *Escherichia coli*  *strain K12* | **B** | 4288 | 3130 | 0.43 | -0.96 | 0.953 | 0.57 | -1.32 | 0.933 |
| *Mycobacterium*  *tuberculosis*  *strain CSU93* | **B** | 3917 | 2857 | 0.43 | -0.94 | 0.935 | 0.57 | -1.27 | 0.922 |
| *Vibrio cholerae*  *strain_N16961* | **B** | 3827 | 2532 | 0.42 | -1.01 | 0.949 | 0.49 | -1.38 | 0.930 |
| *Synechocystis sp*  *strain_PCC6803* | **B** | 3168 | 2171 | 0.24 | -0.97 | 0.942 | 0.32 | -1.31 | 0.929 |
| *Xylella fastidiosa* | **B** | 2765 | 1718 | 0.27 | -1.04 | 0.918 | 0.10 | -1.34 | 0.914 |
| *Thermotoga maritima*  *strain_MSB8* | **B** | 1845 | 1382 | 0.11 | -1.02 | 0.939 | 0.25 | -1.45 | 0.912 |
| *Campylobacter jejuni*  *strain_NCTC11168* | **B** | 1633 | 1212 | -0.04 | -1.07 | 0.964 | -0.08 | -1.42 | 0.907 |
| *Aquifex aeolicus*  *strain_VF5* | **B** | 1521 | 1160 | -0.02 | -1.05 | 0.950 | -0.01 | -1.43 | 0.880 |
| *Chlamydophila pneumoniae*  *strain_J138* | **B** | 1069 | 751 | -0.13 | -1.09 | 0.953 | -0.25 | -1.41 | 0.890 |
| *Chlamydophila pneumoniae*  *strain_CWL029* | **B** | 1051 | 745 | -0.13 | -1.08 | 0.950 | -0.25 | -1.42 | 0.891 |
| *Treponema pallidum*  *strain_Nichols* | **B** | 1030 | 738 | -0.22 | -1.03 | 0.943 | -0.25 | -1.44 | 0.962 |
| *Chlamydophila pneumoniae*  *strain_AR39* | **B** | 996 | 678 | -0.14 | -1.11 | 0.952 | -0.24 | -1.55 | 0.917 |
| *Chlamydia trachomatis strain MoPn* | **B** | 908 | 633 | -0.20 | -1.10 | 0.950 | -0.32 | -1.50 | 0.932 |
| *Chlamydia trachomatis*  *serovar D* | **B** | 893 | 639 | -0.39 | -0.99 | 0.948 | -0.38 | -1.49 | 0.943 |
| *Rickettsia prowazekii*  *strain madridE* | **B** | 833 | 631 | -0.20 | -1.12 | 0.958 | -0.34 | -1.58 | 0.937 |
| *Mycoplasma pneumoniae*  *strain_M129* | **B** | 687 | 491 | -0.31 | -1.17 | 0.908 | -0.64 | -1.66 | 0.920 |
| *Ureaplasma urealyticum*  *strain_serovar3* | **B** | 610 | 382 | -0.52 | -1.12 | 0.982 | -0.83 | -1.54 | 0.901 |
| *Buchnera sp*  *strain_APS* | **B** | 563 | 440 | -0.38 | -1.23 | 0.968 | -0.63 | -1.74 | 0.946 |
| *Mycoplasma genitalium*  *strain_G37* | **B** | 479 | 336 | -0.37 | -1.24 | 0.953 | -0.73 | -1.89 | 0.855 |
|  |  |  |  |  |  |  |  |  |  |
| *Aeropyrum pernix*  *strain_K1* | **A** | 2693 | 1610 | 0.16 | -1.06 | 0.954 | 0.07 | -1.33 | 0.946 |
| *Pyrococcus horikoshii*  *strain_OT3* | **A** | 2063 | 1344 | 0.06 | -1.00 | 0.948 | 0.07 | -1.33 | 0.914 |
| *Methanobacterium*  *thermoautotrophicum*  *strain deltaH* | **A** | 1868 | 1356 | 0.13 | -1.04 | 0.934 | -0.02 | -1.30 | 0.919 |
| *Pyrococcus abyssi*  *strain_GE5* | **A** | 1764 | 1326 | 0.10 | -1.01 | 0.93 | 0.28 | -1.44 | 0.874 |
| *Methanococcus jannaschii*  *strain_DSM2661* | **A** | 1714 | 1209 | -0.04 | -1.04 | 0.958 | 0.04 | -1.37 | 0.882 |
| *Thermoplasma acidophilum* | **A** | 1477 | 1080 | -0.08 | -0.98 | 0.966 | -0.09 | -1.39 | 0.915 |
|  |  |  |  |  |  |  |  |  |  |
| *Eukaryote* |  | 93810 | 56723 | 1.64 | -0.78 | 0.907 | 1.97 | -1.01 | 0.908 |
| *Bacteria* |  | 62499 | 43925 | 1.46 | -0.75 | 0.920 | 1.85 | -0.97 | 0.910 |
| *Archaea* |  | 11579 | 7925 | 0.83 | -0.88 | 0.939 | 0.99 | -1.12 | 0.917 |
|  |  |  |  |  |  |  |  |  |  |
| *All* |  | 167888 | 108573 | 1.71 | -0.71 | 0.904 | 2.14 | -0.92 | 0.901 |

*Labels and abbreviations: SK – superkingdom, ORFs – total number of the open reading frames in the organism’s genome, the column Z>2.9 contains the number of genes with the assigned protein folds.*
