# Supplementary material for: Structural characterization of genomes by large scale sequence-structure threading: application of reliability analysis in structural genomics
Source: BMC Bioinformatics. 2004 Jul 26;5:101. doi: 10.1186/1471-2105-5-101 (PMC499543; doi:10.1186/1471-2105-5-101)
Supplement: Additional File 2 — Parameters α, β and medians (calculated and observed) of Weibull distribution of survival functions of genome occurrences established by maximum likelihood and plotting methods. [file 1471-2105-5-101-S2.doc]

**Table 2. Parameters α, β and medians (calculated and observed) of Weibull distribution of survival functions of genome occurrences** $SDF(GO)=e^{-\left(\frac{GO}{\alpha}\right)^{\beta}}$ established by maximum likelihood and plotting methods.

| ***Organisms*** | **TOPOLOGIES** | | | | | | | | **DOMAINS** | | | | | | | |
| --- | --- | --- | --- | --- | --- | --- | --- | --- | --- | --- | --- | --- | --- | --- | --- | --- |
|  | *Weibull by*  *maximum likelihood* | | | *Weibull by*  *plotting* | | | | *MDN*  *Obsrvd* | *Weibull by*  *maximum likelihood* | | | *Weibull by*  *plotting* | | | | *MDN*  *Obsrvd* |
|  | *α* | *β* | *MDN*  *Calcld.* | *α* | *β* | *r^2^* | *MDN*  *Calcld.* |  | *α* | *β* | *MDN*  *Calcld.* | *α* | *β* | *r^2^* | *MDN*  *Calcld.* |  |
| *Homo sapiens* | 20.40 | 0.60 | 11.12 | 15.00 | 0.50 | 0.974 | 7.16 | 9.00 | 10.77 | 0.70 | 4.00 | 7.30 | 0.51 | 0.983 | 3.58 | 5 |
| *Mus musculus* | 15.12 | 0.63 | 8.47 | 10.93 | 0.51 | 0.978 | 5.32 | 6.00 | 8.64 | 0.76 | 3.38 | 6.07 | 0.56 | 0.989 | 3.15 | 4 |
| *Caenorhabditis elegance* | 15.11 | 0.58 | 8.03 | 9.25 | 0.43 | 0.957 | 3.93 | 5.00 | 9.14 | 0.68 | 2.80 | 5.31 | 0.46 | 0.970 | 2.41 | 4 |
| *Drosophila melanogaster* | 12.96 | 0.61 | 7.09 | 8.14 | 0.46 | 0.970 | 3.65 | 5.00 | 7.73 | 0.72 | 2.74 | 4.75 | 0.50 | 0.983 | 2.28 | 3 |
| *Saccharomyces cerevisiae* | 8.21 | 0.64 | 4.64 | 4.45 | 0.44 | 0.972 | 1.93 | 3.00 | 5.24 | 0.79 | 2.04 | 2.94 | 0.51 | 0.986 | 1.43 | 2 |
| *Plasmodium falciparum* | 4.97 | 0.77 | 3.09 | 3.07 | 0.58 | 0.989 | 1.63 | 2.00 | 3.20 | 0.88 | 2.19 | 1.42 | 0.51 | 0.993 | 0.69 | 1 |
|  |  |  |  |  |  |  |  |  |  |  |  |  |  |  |  |  |
| *Pseudomonas*  *aeruginosa*  *strain PAO1* | 8.20 | 0.64 | 4.64 | 4.56 | 0.45 | 0.963 | 2.03 | 3.00 | 5.28 | 0.80 | 2.09 | 3.15 | 0.54 | 0.990 | 1.60 | 2 |
| *Escherichia coli*  *strain CFT073* | 7.41 | 0.65 | 4.22 | 4.05 | 0.45 | 0.978 | 1.80 | 3.00 | 4.75 | 0.82 | 2.00 | 2.67 | 0.53 | 0.990 | 1.34 | 2 |
| *Escherichia coli*  *strain O157 EDL933* | 7.49 | 0.66 | 4.29 | 4.13 | 0.46 | 0.972 | 1.86 | 3.00 | 4.80 | 0.83 | 2.03 | 2.65 | 0.53 | 0.979 | 1.32 | 2 |
| *Escherichia coli*  *strain O157* | 7.43 | 0.66 | 4.26 | 4.07 | 0.46 | 0.972 | 1.83 | 3.00 | 4.78 | 0.83 | 2.01 | 2.63 | 0.53 | 0.979 | 1.31 | 2 |
| *Salmonella typhimurium*  *strain_LT2* | 6.73 | 0.65 | 3.84 | 3.32 | 0.44 | 0.968 | 1.43 | 3.00 | 4.43 | 0.82 | 1.81 | 2.31 | 0.51 | 0.987 | 1.12 | 2 |
| *Salmonella typhi* | 6.79 | 0.66 | 3.89 | 3.44 | 0.44 | 0.965 | 1.50 | 3.00 | 4.34 | 0.82 | 1.86 | 2.23 | 0.51 | 0.986 | 1.09 | 2 |
| *Escherichia coli*  *strain K12* | 6.88 | 0.66 | 3.96 | 3.58 | 0.45 | 0.971 | 1.59 | 3.00 | 4.49 | 0.84 | 1.89 | 2.46 | 0.53 | 0.989 | 1.24 | 2 |
| *Mycobacterium*  *tuberculosis*  *strain CSU93* | 7.13 | 0.65 | 4.05 | 3.87 | 0.45 | 0.988 | 1.72 | 3.00 | 4.67 | 0.82 | 1.95 | 2.64 | 0.53 | 0.993 | 1.33 | 2 |
| *Vibrio cholerae*  *strain_N16961* | 6.25 | 0.69 | 3.68 | 3.35 | 0.47 | 0.980 | 1.53 | 2.00 | 3.96 | 0.88 | 1.91 | 2.13 | 0.55 | 0.988 | 1.09 | 2 |
| *Synechocystis sp*  *strain_PCC6803* | 5.93 | 0.69 | 3.49 | 2.99 | 0.45 | 0.968 | 1.33 | 2.00 | 3.86 | 0.88 | 1.78 | 1.97 | 0.53 | 0.986 | 0.98 | 2 |
| *Xylella fastidiosa* | 5.20 | 0.72 | 3.13 | 2.69 | 0.48 | 0.985 | 1.25 | 2.00 | 3.21 | 0.90 | 1.84 | 1.43 | 0.50 | 0.991 | 0.69 | 1 |
| *Thermotoga maritima*  *strain_MSB8* | 4.86 | 0.72 | 2.92 | 2.30 | 0.46 | 0.969 | 1.05 | 2.00 | 3.36 | 0.98 | 1.57 | 1.83 | 0.59 | 0.994 | 0.99 | 2 |
| *Campylobacter jejuni*  *strain_NCTC11168* | 4.27 | 0.76 | 2.63 | 1.90 | 0.47 | 0.952 | 0.88 | 2.00 | 2.87 | 1.00 | 1.53 | 1.31 | 0.55 | 0.981 | 0.67 | 1 |
| *Aquifex aeolicus*  *strain_VF5* | 4.43 | 0.75 | 2.72 | 2.02 | 0.48 | 0.949 | 0.94 | 2.00 | 2.95 | 1.00 | 1.56 | 1.45 | 0.57 | 0.980 | 0.76 | 1 |
| *Chlamydophila pneumoniae*  *strain_J138* | 3.70 | 0.82 | 2.36 | 1.62 | 0.48 | 0.982 | 0.76 | 1.00 | 2.62 | 1.06 | 1.44 | 1.20 | 0.57 | 0.987 | 0.63 | 1 |
| *Chlamydophila pneumoniae*  *strain_CWL029* | 3.70 | 0.81 | 2.35 | 1.59 | 0.47 | 0.988 | 0.73 | 1.00 | 2.61 | 1.05 | 1.42 | 1.19 | 0.57 | 0.991 | 0.62 | 1 |
| *Treponema pallidum*  *strain_Nichols* | 3.84 | 0.82 | 2.45 | 1.69 | 0.47 | 0.964 | 0.78 | 2.00 | 2.72 | 1.06 | 1.43 | 1.25 | 0.58 | 0.978 | 0.67 | 1 |
| *Chlamydophila pneumoniae*  *strain_AR39* | 3.57 | 0.83 | 2.29 | 1.55 | 0.48 | 0.985 | 0.73 | 1.00 | 2.50 | 1.11 | 1.42 | 1.16 | 0.60 | 0.994 | 0.63 | 1 |
| *Chlamydia trachomatis strain MoPn* | 3.54 | 0.85 | 2.30 | 1.59 | 0.50 | 0.979 | 0.76 | 2.00 | 2.46 | 1.12 | 1.47 | 1.09 | 0.58 | 0.989 | 0.58 | 1 |
| *Chlamydia trachomatis*  *serovar D* | 3.56 | 0.82 | 2.28 | 1.58 | 0.49 | 0.971 | 0.75 | 2.00 | 2.45 | 1.11 | 1.46 | 1.05 | 0.57 | 0.977 | 0.55 | 1 |
| *Rickettsia prowazekii*  *strain madridE* | 3.52 | 0.86 | 2.29 | 1.58 | 0.51 | 0.968 | 0.77 | 2.00 | 2.36 | 1.15 | 1.50 | 1.05 | 0.60 | 0.996 | 0.57 | 1 |
| *Mycoplasma pneumoniae*  *strain_M129* | 3.00 | 0.88 | 1.99 | 1.23 | 0.50 | 0.971 | 0.59 | 1.00 | 2.04 | 1.28 | 1.36 | 0.83 | 0.62 | 0.986 | 0.46 | 1 |
| *Ureaplasma urealyticum*  *strain_serovar3* | 2.98 | 0.91 | 1.99 | 1.14 | 0.49 | 0.960 | 0.54 | 1.00 | 1.96 | 1.27 | 1.35 | 0.69 | 0.57 | 0.978 | 0.36 | 1 |
| *Buchnera sp*  *strain_APS* | 3.04 | 0.92 | 2.04 | 1.35 | 0.54 | 0.969 | 0.69 | 1.00 | 2.02 | 1.27 | 1.53 | 0.83 | 0.64 | 0.992 | 0.47 | 1 |
| *Mycoplasma genitalium*  *strain_G37* | 2.86 | 0.96 | 1.95 | 1.27 | 0.55 | 0.988 | 0.65 | 1.00 | 1.87 | 1.41 | 1.54 | 0.81 | 0.71 | 0.970 | 0.49 | 1 |
|  |  |  |  |  |  |  |  |  |  |  |  |  |  |  |  |  |
| *Aeropyrum pernix*  *strain_K1* | 4.81 | 0.71 | 2.87 | 2.32 | 0.48 | 0.980 | 1.07 | 2.00 | 3.30 | 0.90 | 1.67 | 1.47 | 0.51 | 0.984 | 0.72 | 2 |
| *Pyrococcus horikoshii*  *strain_OT3* | 4.91 | 0.71 | 2.94 | 2.32 | 0.47 | 0.964 | 1.05 | 2.00 | 3.25 | 0.92 | 1.65 | 1.54 | 0.53 | 0.991 | 0.77 | 1 |
| *Methanobacterium*  *thermoautotrophicum*  *strain deltaH* | 4.81 | 0.72 | 2.90 | 2.35 | 0.48 | 0.975 | 1.09 | 2.00 | 3.23 | 0.95 | 1.66 | 1.54 | 0.53 | 0.983 | 0.77 | 2 |
| *Pyrococcus abyssi*  *strain_GE5* | 4.90 | 0.71 | 2.93 | 2.37 | 0.47 | 0.970 | 1.09 | 2.00 | 3.43 | 0.97 | 1.59 | 1.96 | 0.61 | 0.991 | 1.08 | 2 |
| *Methanococcus jannaschii*  *strain_DSM2661* | 4.30 | 0.72 | 2.59 | 1.83 | 0.45 | 0.965 | 0.82 | 2.00 | 3.06 | 0.94 | 1.47 | 1.43 | 0.53 | 0.986 | 0.72 | 1 |
| *Thermoplasma acidophilum* | 4.59 | 0.74 | 2.81 | 2.04 | 0.46 | 0.955 | 0.91 | 2.00 | 2.91 | 0.98 | 1.56 | 1.34 | 0.55 | 0.982 | 0.68 | 1 |
|  |  |  |  |  |  |  |  |  |  |  |  |  |  |  |  |  |
| *Eukaryote* | 48.80 | 0.55 | 25.15 | 39.50 | 0.49 | 0.971 | 18.76 | 20.00 | 24.11 | 0.62 | 7.02 | 18.29 | 0.50 | 0.982 | 8.77 | 10 |
| *Bacteria* | 45.23 | 0.54 | 23.00 | 35.97 | 0.48 | 0.969 | 16.87 | 20.00 | 25.85 | 0.62 | 6.30 | 20.09 | 0.51 | 0.982 | 9.79 | 12 |
| *Archaea* | 12.50 | 0.60 | 6.80 | 7.87 | 0.46 | 0.964 | 3.57 | 5.00 | 8.11 | 0.73 | 2.66 | 5.09 | 0.51 | 0.974 | 2.47 | 4 |
|  |  |  |  |  |  |  |  |  |  |  |  |  |  |  |  |  |
| *All* | 77.66 | 0.52 | 38.42 | 64.27 | 0.48 | 0.971 | 29.91 | 31.00 | 39.30 | 0.58 | 9.35 | 30.95 | 0.48 | 0.983 | 14.51 | 16 |
