# Supplementary material for: Structural characterization of genomes by large scale sequence-structure threading: application of reliability analysis in structural genomics
Source: BMC Bioinformatics. 2004 Jul 26;5:101. doi: 10.1186/1471-2105-5-101 (PMC499543; doi:10.1186/1471-2105-5-101)
Supplement: Additional File 3 — Statistical parameters for 'Weibull papers' for genomic occurrences of protein topologies and domains. [file 1471-2105-5-101-S3.doc]

Table 3. Statistical parameters for ‘Weibull papers’ for genomic occurrences of protein topologies and domains.

| ***Organisms*** | *Plotting-based Weibull TOPOLOGIES* | | | *Plotting-based Weibull DOMAINS* | | |
| --- | --- | --- | --- | --- | --- | --- |
|  | *63%* | *Exponent Mean*  *Observed* | *α^β^* | *63%* | *Exponent Mean*  *Observed* | *α^β^* |
| *Homo sapiens* | 15.00 | 9.75 | 8.25 | 8.00 | 7.23 | 6.36 |
| *Mus musculus* | 10.66 | 8.90 | 7.43 | 6.00 | 6.65 | 5.95 |
| *Caenorhabditis elegance* | 9.00 | 7.49 | 6.24 | 6.00 | 6.39 | 5.47 |
| *Drosophila melanogaster* | 9.00 | 6.96 | 5.90 | 5.00 | 5.82 | 5.10 |
| *Saccharomyces cerevisiae* | 5.00 | 5.29 | 4.51 | 4.00 | 4.61 | 4.08 |
| *Plasmodium falciparum* | 3.00 | 4.91 | 3.97 | 2.00 | 3.27 | 2.87 |
|  |  |  |  |  |  |  |
| *Pseudomonas*  *aeruginosa*  *strain PAO1* | 5.00 | 5.94 | 4.84 | 4.00 | 4.74 | 4.20 |
| *Escherichia coli*  *strain CFT073* | 4.15 | 5.11 | 4.31 | 3.00 | 4.34 | 3.88 |
| *Escherichia coli*  *strain O157 EDL933* | 5.00 | 5.57 | 4.57 | 3.00 | 4.71 | 4.11 |
| *Escherichia coli*  *strain O157* | 5.00 | 5.54 | 4.55 | 3.00 | 4.71 | 4.11 |
| *Salmonella typhimurium*  *strain_LT2* | 4.00 | 4.89 | 4.07 | 3.00 | 4.15 | 3.66 |
| *Salmonella typhi* | 4.00 | 5.14 | 4.21 | 3.00 | 4.17 | 3.66 |
| *Escherichia coli*  *strain K12* | 4.00 | 5.12 | 4.25 | 3.00 | 4.24 | 3.78 |
| *Mycobacterium*  *tuberculosis*  *strain CSU93* | 4.00 | 4.70 | 4.02 | 3.00 | 4.16 | 3.75 |
| *Vibrio cholerae*  *strain_N16961* | 4.00 | 4.82 | 4.08 | 3.00 | 3.97 | 3.55 |
| *Synechocystis sp*  *strain_PCC6803* | 4.00 | 4.61 | 3.88 | 3.00 | 3.87 | 3.46 |
| *Xylella fastidiosa* | 3.00 | 4.23 | 3.62 | 2.00 | 3.14 | 2.87 |
| *Thermotoga maritima*  *strain_MSB8* | 3.00 | 4.19 | 3.49 | 2.00 | 3.53 | 3.25 |
| *Campylobacter jejuni*  *strain_NCTC11168* | 3.00 | 4.31 | 3.46 | 2.00 | 3.18 | 2.89 |
| *Aquifex aeolicus*  *strain_VF5* | 3.00 | 4.34 | 3.49 | 2.00 | 3.11 | 2.89 |
| *Chlamydophila pneumoniae*  *strain_J138* | 3.00 | 3.43 | 3.00 | 2.00 | 2.79 | 2.64 |
| *Chlamydophila pneumoniae*  *strain_CWL029* | 2.00 | 3.24 | 2.88 | 2.00 | 2.74 | 2.60 |
| *Treponema pallidum*  *strain_Nichols* | 3.00 | 3.66 | 3.16 | 2.00 | 3.22 | 2.90 |
| *Chlamydophila pneumoniae*  *strain_AR39* | 2.00 | 3.27 | 2.90 | 2.00 | 2.77 | 2.62 |
| *Chlamydia trachomatis strain MoPn* | 2.00 | 3.55 | 3.05 | 2.00 | 2.79 | 2.62 |
| *Chlamydia trachomatis*  *serovar D* | 2.00 | 3.40 | 2.98 | 2.00 | 2.92 | 2.68 |
| *Rickettsia prowazekii*  *strain madridE* | 2.00 | 3.70 | 3.13 | 2.00 | 2.73 | 2.56 |
| *Mycoplasma pneumoniae*  *strain_M129* | 2.00 | 3.02 | 2.65 | 1.00 | 2.47 | 2.36 |
| *Ureaplasma urealyticum*  *strain_serovar3* | 2.00 | 3.31 | 2.79 | 1.00 | 2.27 | 2.19 |
| *Buchnera sp*  *strain_APS* | 2.00 | 3.43 | 2.89 | 1.00 | 2.50 | 2.33 |
| *Mycoplasma genitalium*  *strain_G37* | 2.00 | 3.08 | 2.70 | 1.00 | 2.26 | 2.19 |
|  |  |  |  |  |  |  |
| *Aeropyrum pernix*  *strain_K1* | 3.00 | 4.40 | 3.53 | 2.00 | 3.44 | 3.06 |
| *Pyrococcus horikoshii*  *strain_OT3* | 3.00 | 4.34 | 3.53 | 2.00 | 3.27 | 2.97 |
| *Methanobacterium*  *thermoautotrophicum*  *strain deltaH* | 3.00 | 4.12 | 3.43 | 2.00 | 3.40 | 3.09 |
| *Pyrococcus abyssi*  *strain_GE5* | 3.00 | 4.23 | 3.48 | 2.00 | 3.67 | 3.36 |
| *Methanococcus jannaschii*  *strain_DSM2661* | 2.00 | 3.98 | 3.22 | 2.00 | 3.07 | 2.83 |
| *Thermoplasma acidophilum* | 3.00 | 4.35 | 3.54 | 2.00 | 3.29 | 2.93 |
|  |  |  |  |  |  |  |
| *Eukaryote* | 36.00 | 15.23 | 12.78 | 18.00 | 10.71 | 9.39 |
| *Bacteria* | 31.20 | 13.54 | 11.37 | 21.00 | 10.59 | 9.45 |
| *Archaea* | 9.00 | 7.47 | 6.04 | 6.00 | 6.25 | 5.44 |
|  |  |  |  |  |  |  |
| *All* | 53.00 | 17.63 | 14.80 | 31.00 | 12.58 | 11.09 |

Here the columns marked ‘63%’ contain the estimated values of the Weibull characteristic life at 63% of the distributions, ‘*Exponent Mean Observed*’ correspond to the actual mean values for the distributions and parameters *α^β^* have been computed from the analytical β and α Weibull coefficients.
